# GADD45α and γ interaction with CDK11p58 regulates SPDEF protein stability and SPDEF-mediated effects on cancer cell migration

**DOI:** 10.18632/oncotarget.7355

**Published:** 2016-02-12

**Authors:** Rodrigo E. Tamura, Juliano D. Paccez, Kristal C. Duncan, Mirian G. Morale, Fernando M. Simabuco, Simon Dillon, Ricardo G. Correa, Xuesong Gu, Towia A. Libermann, Luiz F. Zerbini

**Affiliations:** ^1^ International Centre for Genetic Engineering and Biotechnology (ICGEB), Medical Biochemistry Division, Faculty of Health Sciences, University of Cape Town, Werner and Beit Building South, Cape Town, South Africa; ^2^ BIDMC Genomics, Proteomics, Bioinformatics and Systems Biology Center, Beth Israel Deaconess Medical Center and Harvard Medical School, Boston, MA, USA; ^3^ Sanford Burnham Prebys Medical Discovery Institute, La Jolla, CA, USA

**Keywords:** CDK11p58, SPDEF, GADD45, migration, invasion

## Abstract

The epithelium-specific Ets transcription factor, SPDEF, plays a critical role in metastasis of prostate and breast cancer cells. While enhanced SPDEF expression blocks migration and invasion, knockdown of SPDEF expression enhances migration, invasion, and metastasis of cancer cells. SPDEF expression and activation is tightly regulated in cancer cells; however, the precise mechanism of SPDEF regulation has not been explored in detail. In this study we provide evidence that the cell cycle kinase CDK11p58, a protein involved in G2/M transition and degradation of several transcription factors, directly interacts with and phosphorylates SPDEF on serine residues, leading to subsequent ubiquitination and degradation of SPDEF through the proteasome pathway. As a consequence of CDK11p58 mediated degradation of SPDEF, this loss of SPDEF protein results in increased prostate cancer cell migration and invasion. In contrast, knockdown of CDK11p58 protein expression by interfering RNA or SPDEF overexpression inhibit migration and invasion of cancer cells. We demonstrate that CDK11p58 mediated degradation of SPDEF is attenuated by Growth Arrest and DNA damage-inducible 45 (GADD45) α and, two proteins inducing G2/M cell cycle arrest. We show that GADD45 α and γ, directly interact with CDK11p58 and thereby inhibit CDK11p58 activity, and consequentially SPDEF phosphorylation and degradation, ultimately reducing prostate cancer cell migration and invasion. Our findings provide new mechanistic insights into the complex regulation of SPDEF activity linked to cancer metastasis and characterize a previously unidentified SPDEF/CDK11p58/GADD45α/γ pathway that controls SPDEF protein stability and SPDEF-mediated effects on cancer cell migration and invasion.

## INTRODUCTION

Metastatic prostate cancer lacks any effective therapy, although clinical efficacy of the first anti-metastatic drug cabozantinib has shown some promise [1]. An impediment to improve outcome is lack of thorough understanding of the underlying molecular mechanisms. Deciphering key molecular mechanisms and drivers of prostate cancer metastasis will enable identification of new therapeutic targets and avenues, providing for critical advancement in prostate cancer treatment. The discovery and functional validation of recurrent gene fusions of 5 Ets factor genes (ETV1, ETV4, ETV5, ERG, Fli1) in prostate cancer highlights the relevance of Ets transcription factors as oncogenes in prostate cancer [2-4]. The potential role of the non-fusion epithelial-specific Ets factor, SPDEF, as prostate cancer tumor and metastasis suppressor recently emerged, demonstrating that SPDEF expression loss in advanced prostate cancer correlates with poor outcome [5-11]. Thus, downregulation of SPDEF expression may be critical for prostate cancer progression. Indeed, we and others have shown that inhibition of SPDEF expression in prostate cancer cells reduces adhesion and increases migration, invasion and EMT, while overexpression prevents these functions indicating a role of SPDEF in prostate cancer metastasis, but with apparent opposite activity to ERG [5, 6, 12]. The role of SPDEF in metastasis is further supported by the recent study showing that SPDEF knockdown in prostate cancer cells enhances and overexpression reduces survival of prostate cancer cells at distal sites and metastasis formation [11]. SPDEF acts as a tumor suppressor in colorectal cancer through the E-cadherin and β-catenin pathway and may play similar roles in a number of other epithelial cancers [5, 13-15]. While we have demonstrated interaction of SPDEF with the androgen receptor (AR) and various other proteins relevant for cancer signalling [5, 16], no studies have been reported as of yet about the precise regulatory mechanisms of SPDEF protein expression and activity in the context of metastasis.

In this study we sought to gain new insights into the role phosphorylation and protein degradation plays in SPDEF-dependent effects on prostate cancer cell migration and invasion. Our hypothesis is that a shift in SPDEF protein turnover is a key step in prostate cancer metastasis and that degradation of SPDEF initiates a cascade of events associated with metastatic spread of prostate cancer.

## RESULTS

### GADD45α, like SPDEF, blocks migration and invasion of prostate cancer cells

We previously demonstrated that SPDEF effectively inhibits prostate cancer migration and invasion and may, thus, function as a tumor and metastasis suppressor [5]. Likewise, our data provided strong evidence that reduced SPDEF expression induces migration, invasion and EMT. We also showed that GADD45α and γ induce cell cycle arrest and apoptosis in prostate cancer cells, and NF-κB- as well as JunD-mediated repression of GADD45α and γ are essential for prostate cancer cells to escape cell death [17, 18]. GADD45α expression is repressed due to methylation [17], and reduced SPDEF expression directly correlates with poor outcome in prostate cancer [7, 9]. A review of published gene expression data, moreover, suggests that regulation of SPDEF expression correlates with GADD45α in LNCaP prostate cancer and MCF-7 breast cancer cells. Based on these findings we tested the hypothesis that SPDEF and GADD45α and γ are functionally interconnected and overlap in their tumor suppressive roles in prostate cancer.

**Figure 1:**
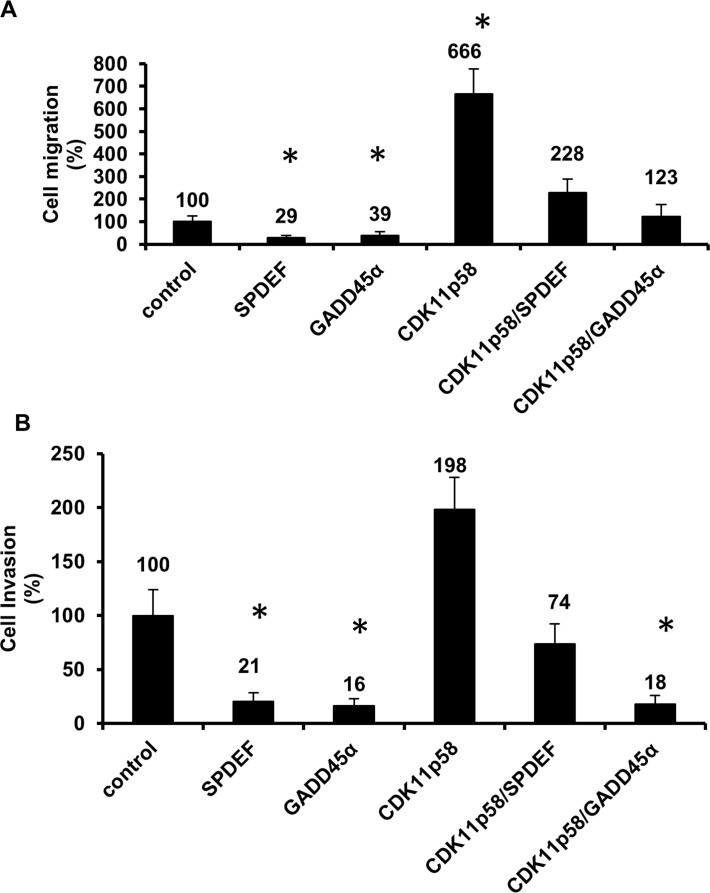
CDK11p58 and GADD45α affect prostate cancer cell migration and invasion DU145 were transfected with SPDEF-Flag, GADD45α-Flag, CDK11p58-HA, CDK11p58-HA/SPDEF-Flag, CDK11p58-HA/GADD45 α-Flag or mock transfected. **A.** Migration and **B.** invasion were measured using transwells 24h post-transfection. Cells were fixed and stained, and 8-10 random microscopic fields were counted and the mean number of the control cells was considered as 100%, and the experimental groups are shown as relative to the control. Student-*t* test was used to evaluate the statistical difference among the groups compared to the control * indicates statistical significance with *p*-value < 0.0001.

We evaluated the impact of SPDEF and GADD45α on migration and invasion of DU145 prostate cancer cells. As previously reported [5], SPDEF reduced both migration and invasion and GADD45α likewise inhibited migration and invasion at a similar rate as SPDEF (Figure 1A-1B and see Additional file 1: Figure S1).

### All three GADD45 family members interact with the cell cycle kinase CDK11p58

**Figure 2:**
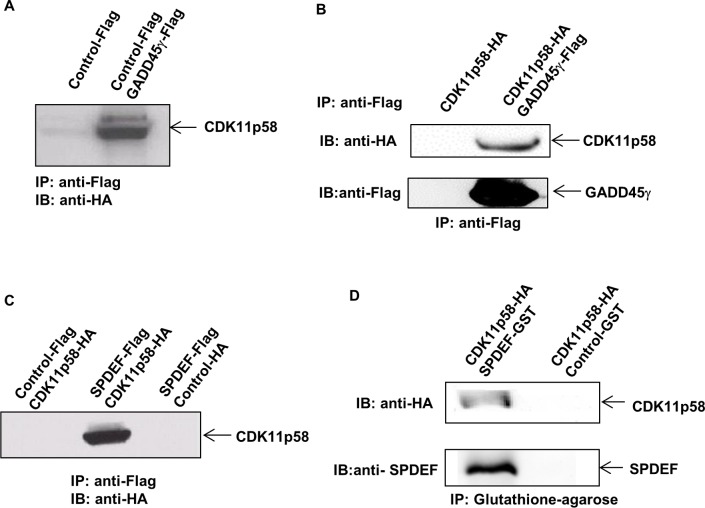
Interaction of SPDEF, CDK11p58 and GADD45γ **A.** DU145 cells were transfected with GADD45γ-Flag and CDK11p58-HA. Protein extracts were immunoprecipitated using anti-FLAG mAb and interaction with CDK11p58 was detected by anti-HA antibody. **B.** CDK11p58 interacts directly with GADD45γ. GADD45γ-Flag and CDK11p58-HA proteins were *in vitro* translated and immunoprecipitated using anti-FLAG mAb. Interaction with CDK11p58 was detected by anti-HA antibody. **C.** CDK11p58 interacts with SPDEF. DU145 cells were transfected with SPDEF-Flag and CDK11p58-HA. Protein extracts were immunoprecipitated using anti-FLAG mAb. Interaction with CDK11p58 was detected by anti-HA antibody. **D.** Direct interaction between CDK11p58 and SPDEF. GST and SPDEF-GST proteins were incubated with *in vitro* translated CDK11p58 and immunoprecipitated using glutathione-agarose beads and after SDS-PAGE immunoblotted with anti-HA and anti-PDEF antibodies.

Our previous mass spectrometric approach identified interaction partners of GADD45 proteins *in vivo* using HEK 293T cells, resulting in identification of MEKK4, the upstream kinase of JNK, as an *in vivo* interactor of all three members of the GADD45 family (α, β and γ) [18]. Here, we further explored these mass spectrometric data from the pull-down experiment and identified the CDK11 protein family member, CDK11p58, as a new *in vivo* interactor of GADD45γ (see Additional file 2: Figure S2). To validate this result, DU145 cells were transiently transfected with the GADD45γ-Flag expression vector or parental Flag vector as control. Immunoprecipitation was performed to pull down GADD45-interacting proteins using anti-Flag antibody. Western blot analysis of the immunoprecipitated proteins using anti-CDK11p58 antibody showed that CDK11p58 specifically interacts with GADD45γ (Figure 2A). To further confirm this interaction, the proteins were expressed *in vitro* in a cell-free system and the translated GADD45γ-Flag and CDK11p58-HA proteins were either combined or kept separate. Immunoprecipitation using anti-Flag-agarose beads followed by Western blot analysis using anti-HA antibody confirmed that CDK11p58 directly binds to GADD45γ, since CDK11p58 was only pulled down in the presence of GADD45γ (Figure 2B).

### CDK11p58directly interacts with SPDEF

Since reduced SPDEF expression/activity is a key driver of metastasis in prostate cancer, we tested our hypothesis that the interplay between GADD45α and CDK11p58 elicits its effects on prostate cancer migration and invasion through modulation of SPDEF expression or activity. We tested whether SPDEF interacts with CDK11p58 and/or GADD45α. We co-transfected DU145 cells with CDK11p58-HA and SPDEF-Flag or Flag expression vectors and performed immunoprecipitation using anti-Flag antibody followed by Western blot analysis with anti-HA antibody. As shown in Figure 2C, CDK11p58 co-immunoprecipitates with SPDEF, but not with Flag. To evaluate whether CDK11p58-SPDEF interaction is direct, we expressed the SPDEF-GST fusion protein in a bacterial system. GST-pull down confirmed that CDK11p58-HA directly interacts with SPDEF (Figure 2D).

### GADD45α inhibits CDK11p58-mediated increase in migration and invasion

**Figure 3:**
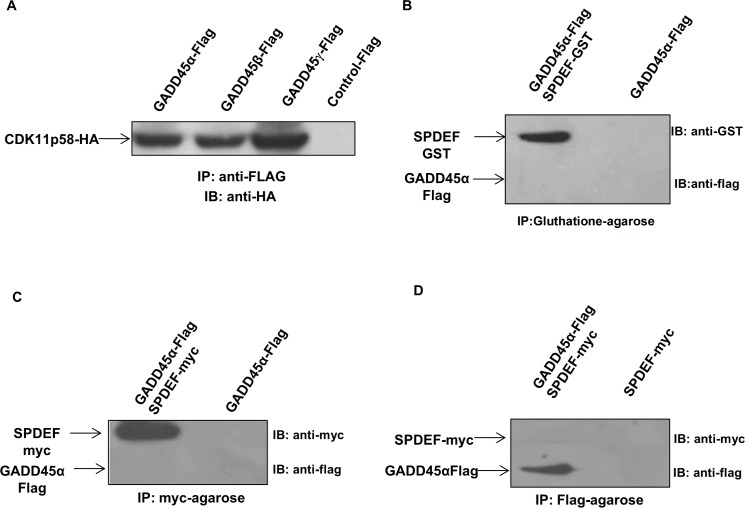
Interaction of SPDEF, GADD45 and CDK11p58 proteins **A.** CDK11p58 binds to the GADD45 family genes. HEK293T cells were transfected with GADD45α-Flag, GADD45β-Flag, GADD45γ-Flag, and CDK11p58-HA constructs. Protein extracts were immunoprecipitated using anti-FLAG mAb antibody, and the presence of interaction with CDK11p58 were probed by anti-HA antibody. **B.** GADD45 does not interact with SPDEF. SPDEF-GST protein was expressed in BL21 bacteria cells followed by GST-pull down assay and GADD45 protein was *in vitro* translated using TNT^®^ *Quick* Coupled transcription/translation system (Promega). GST and SPDEF-GST proteins were incubated with *in vitro* translated GADD45 protein for 16 hours and subjected to SDS-PAGE gel. Immunoblot was performed using anti-GST and anti-flag antibodies, (C and D)GADD45 does not interact with SPDEF. SPDEF-myc and GADD45 α-Flag proteins were *in vitro* translated using TNT ^®^ *Quick* Coupled transcription/translation system (Promega), immunoprecipitated using anti Myc-agarose or anti Flag-agarose and subjected to SDS-PAGE gel. The detection of SPDEF-myc protein was performed using anti-myc antibodies, while GADD45α-Flag was detected using anti-FLAG antibodies.

We evaluated whether all three GADD45 family proteins, GADD45α, GADD45β and GADD45γ, bind to CDK11p58. HEK 293T cells were transfected with expression vectors for the GADD45-Flag family members and CDK11p58-HA. Immunoprecipitation using anti-Flag antibody followed by Western blot analysis using anti-HA antibody demonstrated that CDK11p58 interact with GADD45α, β and γ proteins (Figure 3A).

To investigate the impact of interaction between GADD45α and CDK11p58 on CDK11p58 function, we evaluated the effect of transiently transfected CDK11p58 on migration and invasion of DU145 cells. In contrast to SPDEF and GADD45α, CDK11p58 strongly increased prostate cancer migration and invasion (Figure 1A-1B). However, co-transfection with CDK11p58 and GADD45α significantly reduced CDK11p58-dependent migration and invasion (Figure 1A-1B), suggesting that GADD45α interaction with CDK11p58 attenuates the pro-metastatic activity of CDK11p58.

The impact of CDK11p58 on migration and invasion was confirmed in DU145 cells infected with a lentivirus encoding shRNA against CDK11p58 (DU145-CDK11p58−/−) or GFP (DU145GFP−/−). Analysis of a wound-healing assay in DU145GFP−/− and DU145CDK11p58−/− cell lines showed a slower rate of migration of DU145CDK11p58−/− cells as compared to the control DU145GFP−/− cells (see Additional file 1: Figure S1C).

In addition, GST pull down of SPDEF-GST and GADD45α-Flag did not shown any direct interaction (Figure 3B). This result was further corroborated by immunoprecipitation of *in vitro* translated SPDEF-myc and GADD45α-Flag proteins using anti-myc agarose or anti-Flag agarose (Figure 3C-3D). Thus, SPDEF, GADD45α and GADD45γ directly interact with CDK11p58, but not with each other.

### CDK11p58 phosphorylates SPDEF

The nuclear Ser/Thr kinase CDK11p58 is involved in degradation of several transcriptional factors such as Estrogen receptor α (ERα) and AR [19-21]. We evaluated whether, as a consequence of CDK11p58 interaction with SPDEF, CDK11p58 phosphorylates SPDEF. An *in vitro* kinase assay with SPDEF in the presence or absence of CDK11p58 followed by Western blot analysis using monoclonal antibodies against phospho-Serine (pSer) demonstrated phosphorylation of SPDEF on serine residues only in the presence of CDK11p58 (see Additional file 3: Figure S3).

### GADD45α inhibits CDK11p58 kinase activity

**Figure 4:**
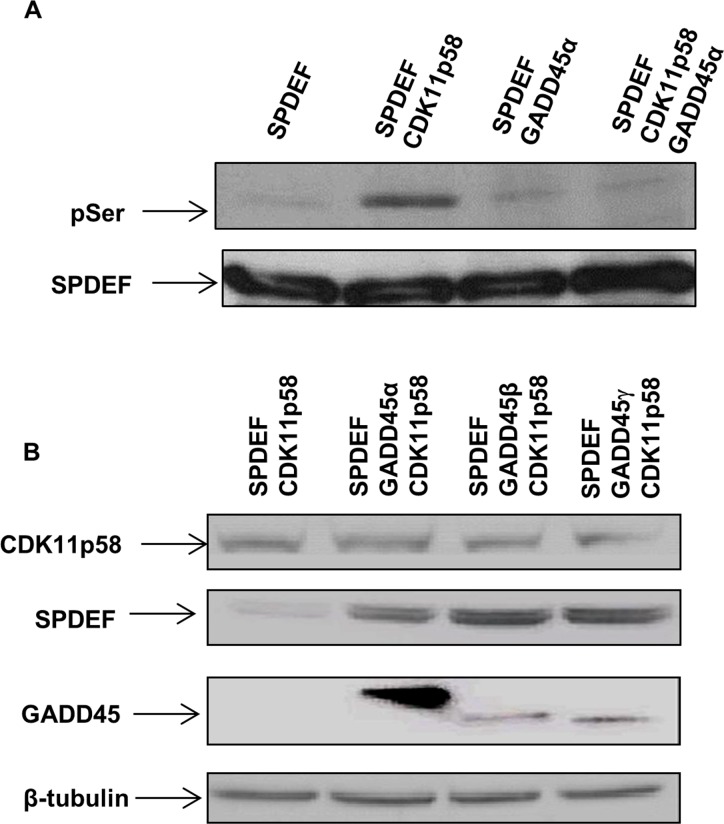
GADD45 inhibits CDK11p58 kinase activity and SPDEF degradation **A.** Phosphorylation of SPDEF by CDK11p58 is inhibited by GADD45 proteins. SPDEF, GADD45 and CDK11p58 were produced *in vitro* by TNT^®^ *Quick* Coupled transcription/translation system (Promega) and combinations of SPDEF and mock or SPDEF and GADD45 were subjected to *in vitro* kinase assay mediated by CDK11p58. SPDEF was detected using anti-flag and phosphorylated SPDEF was detected with anti-serine antibody. **B.** CDK11p58-mediated degradation of SPDEF is blocked by GADD45. Western blot of HEK 293T cells transfected with SPDEF-Flag and CDK11p58-HA in absence or presence of GADD45α-Flag, GADD45β-Flag or GADD45γ-Flag using anti-Flag and anti-HA antibodies.

GADD45 family members interact with various signaling kinases [22, 23]. In some instances GADD45 proteins activate the interacting kinase such as MTK1/MEKK4, whereas interaction of all three GADD45 family members with CDK1 inhibits CDK1 kinase activity [23]. To determine whether GADD45α enhances or decreases CDK11p58 kinase activity, an *in vitro* CDK11p58 kinase assay with SPDEF as a substrate was performed. No significant serine phosphorylation on SPDEF occurred when only GADD45α was present. CDK11p58 induced SPDEF phosphorylation which was completely inhibited by GADD45α indicating that GADD45α blocks the kinase activity of CDK11p58 (Figure 4A).

To determine whether inhibition of CDK11p58 kinase activity by GADD45α prevents CDK11p58-mediated SPDEF degradation, the effect of GADD45α on SPDEF degradation was tested in HEK 293T cells in the presence of CDK11p58. Western blot analysis for SPDEF revealed that overexpression of GADD45α, β and γ prevents SPDEF degradation mediated by CDK11p58 (Figure 4B). These data confirm that CDK11p58 regulation of SPDEF protein stability is controlled by GADD45 and that shifts in the levels of GADD45 and CDK11p58 proteins may play an important role in prostate cancer migration and invasion.

### CDK11p58 induces degradation of SPDEF protein

**Figure 5:**
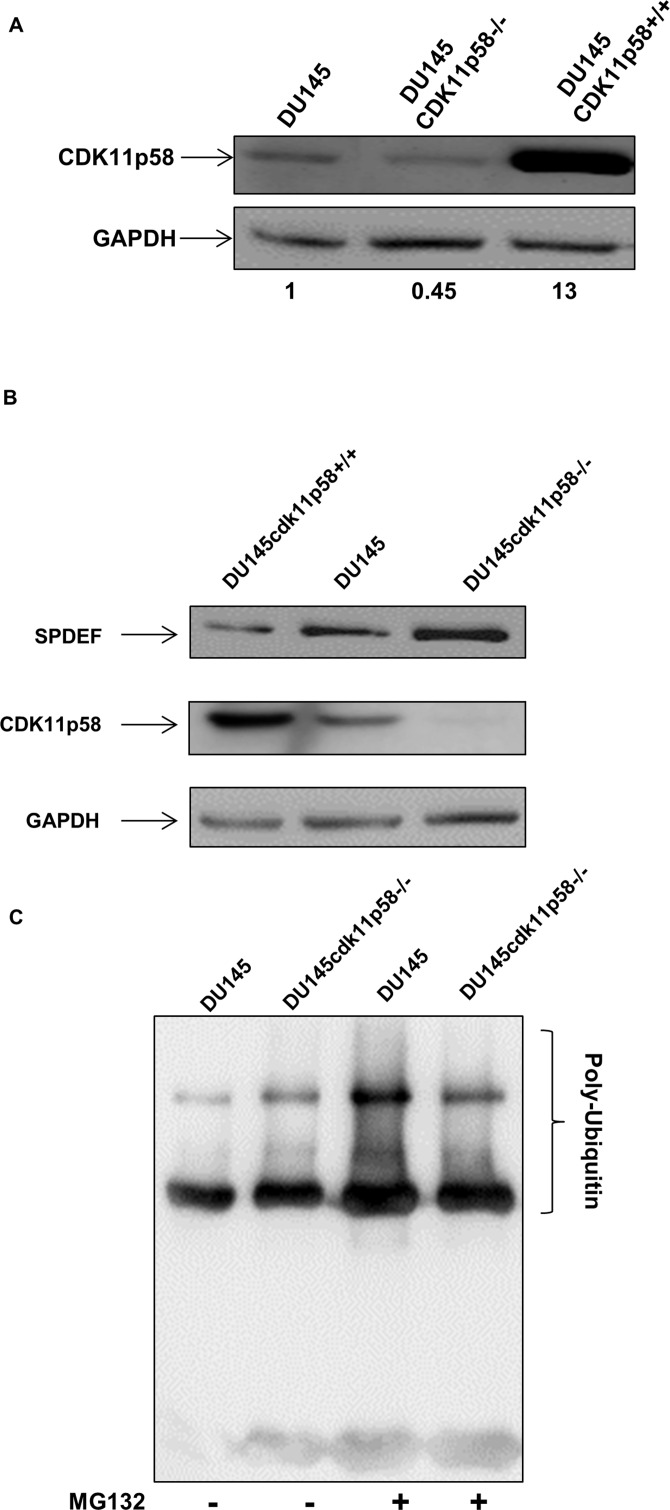
Degradation of SPDEF mediated by CDK11p58 **A.** Differential expression of CDK11p58 expression in DU145-CDK11p58−/−, DU145-CDK11p58+/+ and DU145 cell lines. Whole cell extract was subjected to SDS-Page and immunoblot performed using anti-HA and anti-GAPDH (Bio Rad) antibodies. **B.** Differential expression of SPDEF. DU145-CDK11p58−/−, DU145-CDK11p58+/+ and DU145 cell lines were transfected with CDK11p58-HA and SPDEF-flag vectors and protein were analysed by western-blot. Detection of CDK11p58 by anti HA-HRP antibody (Sigma), anti-SPDEF and anti-GAPDH (Bio Rad) antibodies. **C.** Ubiquitination of SPDEF is mediated by CDK11p58. DU145 and DU145-CDK11p58−/− cells were transfected with SPDEF-Flag and Ubiquitin-myc vectors. 24 hours after transfection, cells were treated with MG132 for 4 hours and 500μg of total protein were immunoprecipitated using anti-Flag antibody and ubiquitin was detected with anti-myc antibody.

CDK11p58 induces ubiquitin/proteasome dependent protein degradation of several transcription factors [20, 21, 24]. To gain insights into the functional consequences of CDK11p58-mediated phosphorylation of SPDEF, we applied gain-of-function and loss-of-function of CDK11p58 assays. We developed DU145 cell lines either overexpressing CDK11p58 protein (DU145-CDK11p58+/+) or knocking down CDK11p58 protein expression (DU145-CDK11p58−/−). DU145-CDK11p58+/+ cells showed a 13 fold increase in CDK1158 expression and DU145-CDK11p58−/− cells had a 55% decrease in CDK11p58 expression (Figure 5A). Western blot analysis of endogenous SPDEF of DU145-CDK11p58−/− and DU145-CDK11p58+/+ cells revealed a 43% decrease of SPDEF protein in DU145-CDK11p58+/+ cells and a 1.8 fold increase in DU145-CDK11p58−/− cells when compared to parental DU145 cells (Figure 5B), corroborating that CDK11p58-mediated phosphorylation of SPDEF induces SPDEF degradation. Immunoprecipitation of SPDEF-Flag in DU145 and DU145-CDK11p58−/− in the presence of ubiquitin-myc with and without the proteasome inhibitor MG132, which inhibits proteasome mediated degradation of ubiquitinated proteins, followed by Western blot analysis with anti-myc antibody demonstrates that SPDEF is poly-ubiquitinated and degraded by the proteasome pathway (Figure 5C), since MG132 increase poly-ubiquinated SPDEF protein levels. In contrast, in CDK11p58 knockdown cells, the increase in SPDEF ubiquitination upon MG132 treatment is reduced (Figure 5C). These findings indicate that CDK11p58 induces SPDEF degradation through the ubiquitination pathway.

### SPDEF, CDK11p58, and ubiquitin co-localize in the nucleus

**Figure 6:**
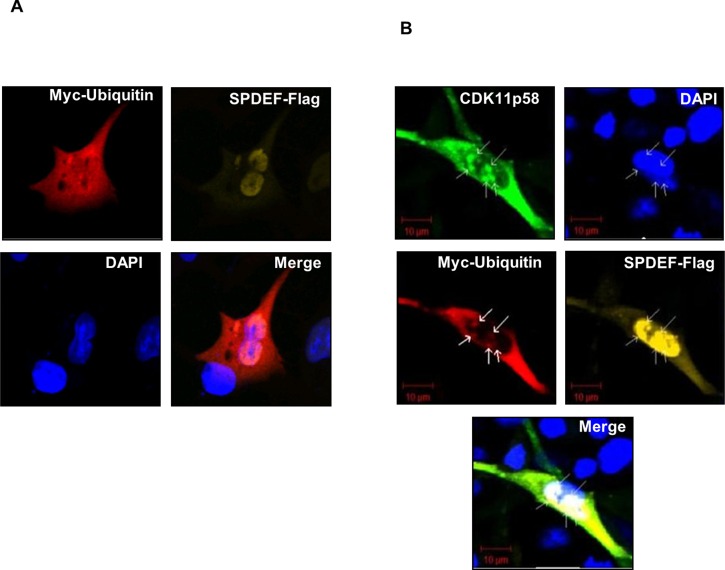
Co-localization of SPDEF, CDK11p58 and ubiquitin DU145 cells were transfected with **A.** SPDEF-Flag and Ubiquitin-myc and **B.** CDK11p58-HA, Ubiquitin-myc and SPDEF-Flag vectors. 24 hours after transfection cells were treated with MG132 for 4 hours, fixed, and incubated for 1 hour with antibodies against HA-FITC, Myc-Cy3 and against Flag produced in rabbit and then incubated for 1 hour with antibody against rabbit conjugated with Cy5 and DAPI. Cells were analysed by Confocal Laser Scanning Microscope (Carl Zeiss). Magnification, X 200.

To establish the cellular location of interaction between CDK11p58 and SPDEF, SPDEF-Flag and Ubiquitin-myc expression vectors were transfected into DU145 cells in the absence or presence of the CDK11p58-HA expression vector. Confocal laser microscopy of transfected cells revealed that SPDEF-Flag is localized in the nucleus, while Ubiquitin-myc is distributed in the cytoplasm and the nucleus and does not specifically co-localize with SPDEF (Figure 6A). Co-transfection of CDK11p58 results in a clear co-localization of CDK11p58, SPDEF and Ubiquitin-myc in clusters inside the nucleus (Figure 6B). Similar analysis indicates that GADD45α and CDK11p58 co-localize in both the nucleus and cytoplasm (see Additional file 4: Figure S4).

### Mutation analysis of SPDEF identifies phosphorylation sites for CDK11p58 involved in protein degradation

**Figure 7:**
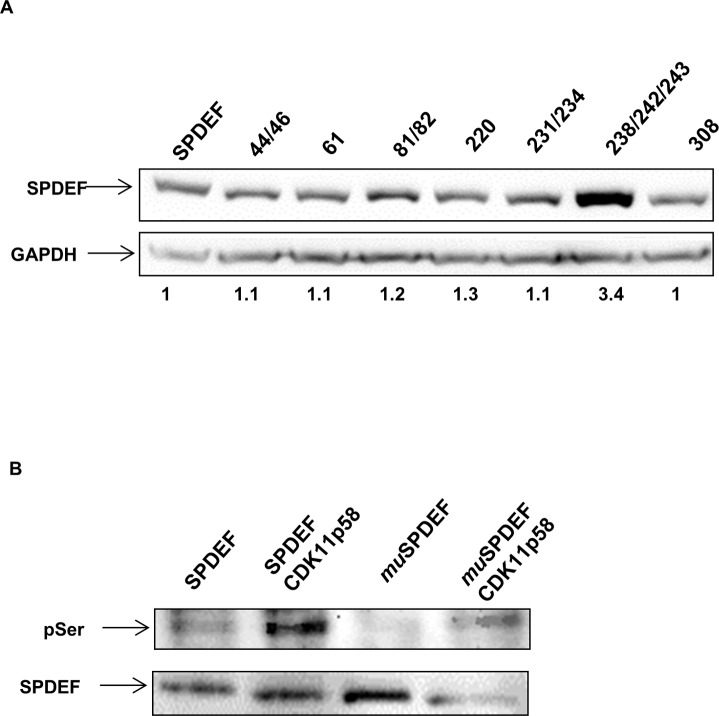
SPDEF mutational analysis **A.** Western-blot analysis of SPDEF and SPDEF mutant proteins. SPDEF and SPDEF mutant plasmids were transfected in HEK 293T and immunoblotted using anti-SPDEF antibody. **B.** *In vitro* kinase assay of SPDEF and SPDEF mutated in the residues 238/242/243 by CDK11p58 and detection using anti p-serine antibody.

Candidate SPDEF phosphorylation sites were predicted by the NetPhos2.0 server. To identify which sites are responsible for SPDEF phosphorylation and consequent degradation by CDK11p58 we replaced high scoring candidate serine phosphorylation sites of SPDEF with alanine. Some of these mutants contained more than one mutation (S44A/S46A, S61A, S61A/S62A, S220A, S231A/S234A, S238A/S242A/S243A, S308A). We co-transfected HEK 293T cells with CDK11p58-HA plasmid together with wild type or mutant SPDEF plasmids. Western blot analysis demonstrated that only one of the mutant constructs, consisting of mutations of serine 238, 242 and 243, resulted in increased levels of SPDEF protein expression as compared to wild type SPDEF (Figure 7A), indicating that alterations of the serine residues 238, 242 and/or 243 may increase protein stability and may be the targets of CDK11p58 kinase activity. An *in vitro* kinase assay comparing phosphorylation of SPDEF*wt* to mutated SPDEF-S238A/S242A/S243A (SPDEF*mu*) supported this conclusion, since levels of phosphorylation of this mutant were reduced in the presence of CDK11p58 as compared to wild type SPDEF (Figure 7B).

### CDK11p58 counteracts the metastasis suppressor activity of SPDEF

To further confirm that CDK11p58 enhances migration and invasion of prostate cancer cells by inducing degradation of SPDEF, a critical step in prostate cancer metastasis, we measured migration and invasion of DU145 cells overexpressing SPDEF in the absence or presence of CDK11p58. While SPDEF suppressed migration and invasion, CDK11p58 completely reversed the metastasis suppressive function of SPDEF, most likely as a consequence of CDK11p58-mediated SPDEF degradation (Figure 1A-1B).

**Figure 8:**
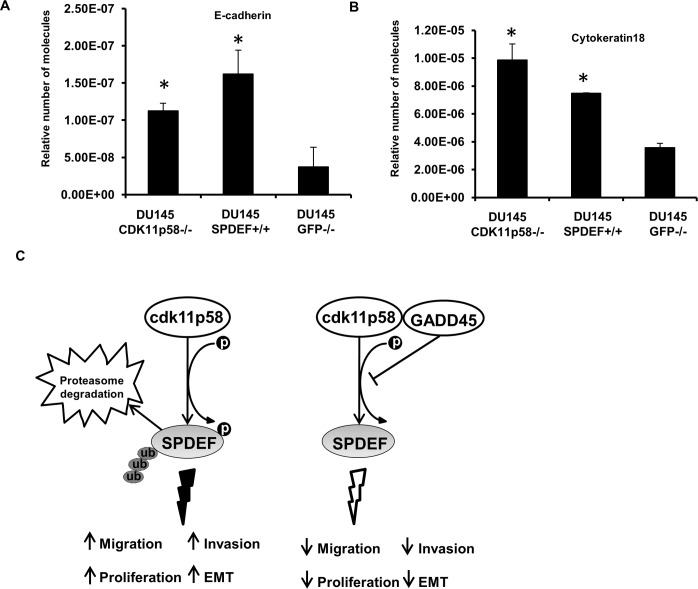
SPDEF induces expression of genes associated with epithelial phenotype Real-time PCR for Cytokeratin-18 **A.** and E-cadherin **B.** in DU145GFP−/−, DU145-CDK11p58−/− and DU145-SPDEF+/+ cells. Student-*t* test was used to evaluate the statistical difference among the groups compared to the control. * indicates statistical significance with *p*-value < 0.01. **C.** Schematic representation of SPDEF regulation by CDK11p58 in prostate cancer cells.

Deregulation of various Ets factors leads to altered expression of key genes required for cancer progression. SPDEF has been previously shown to act as a negative regulator of tumor progression and to elicit its metastasis suppressor function by regulating a gene expression program linked to maintaining the epithelial phenotype, while knock down of SPDEF induces EMT by reducing expression of epithelial-specific genes such as E-cadherin and cytokeratin 18 and enhancing expression of mesenchymal genes that promote prostate cancer migration, invasion and metastasis [5, 13-15, 25]. Indeed SPDEF directly transactivates the E-cadherin gene in prostate cancer [10]. We measured expression of two SPDEF target genes, cytokeratin 18 and E-cadherin, by real-time PCR in DU145 knockdown CDK11p58 (DU145-CDK11p58−/−) and SPDEF-overexpressing DU145 cells as compared to DU145 cells expressing siRNA against GFP (DU145-GFP−/−). Compared to control cells (DU145-GFP−/−), overexpression of SPDEF induced cytokeratin 18 and E-cadherin expression (Figure 8A). The same upregulation of cytokeratin 18 and E-cadherin expression was obtained when CDK11p58 expression was knocked down (Figure 8B), further supporting our conclusions that CDK11p58 impacts prostate cancer cell migration and invasion through regulation of SPDEF protein stability. Similar effects of CDK11p58 and SPDEF on Cytokeratin 18 and E-cadherin expression were seen in another prostate cancer cell line, PC-3 (data not shown).

## DISCUSSION

We have identified an intricate pathway of SPDEF regulation that involves CDK11p58-dependent phosphorylation of SPDEF on amino acids residues 238, 242 and/or 243, resulting in SPDEF ubiquitination and degradation through the proteasome pathway, and inhibition of CDK11p58 activity by direct interaction with GADD45α and other GADD45 family members, which induce apoptosis in prostate cancer cell lines [18] and are repressed by promoter methylation in PCa [26] Thereby, the precise level of SPDEF protein and the actual metastasis-suppressive or -promoting activity can be tightly regulated by unique and dynamic signaling inputs.

We demonstrate that CDK11p58 directly interacts with SPDEF. The CDK11 protein family is encoded by two nearly identical genes, CDC2L1 and CDC2L2, and forms three main isoforms, CDK11p110, CDK11p58, and CDK11p46 [27]. CDK11p58 inhibits p21 activated kinase 1 (PAK1) [20], ERα [28], vitamin D receptor (VDR) [19] and AR [21] by inducing their phsophorylation and degradation through the proteasome pathway. Under normal physiological conditions CDK11p58 is primarily expressed during G2/M, is essential for cell cycle progression during mitosis and has been linked to tumorigenesis, although the precise role in cancer remains unknown [29, 30]. In osteosarcoma CDK11 is overexpressed and correlates with poor outcome [31]. CDK11 is critical for proliferation and escape from programmed cell death [31]. Overexpression of CDK11p58 is observed in pancreatic cancer cells and multiple myeloma, promoting proliferation and protecting cells from programed cell death [32]. With regard to prostate cancer, one report suggests that CDK11p58 expression is reduced in primary prostate cancer tissue; however, neither kinase activity nor level of activation of CDK11p58 in metastatic prostate cancer was tested [24].

Oncogenic activities of Ets fusion proteins in prostate cancer have been studied in detail; however, potential tumor and metastasis suppressor activities of Ets factors have been neglected until recently, when we and others showed that SPDEF is a tumor and metastasis suppressor in prostate cancer [5, 11]. Metastasis, the main cause of mortality in prostate cancer, involves changes in adhesion, migration and invasion and likely includes EMT-like properties, a switch from an epithelial to a mesenchymal gene expression program [33]. Although the EMT-like phenotype plays a critical role in progression and metastasis of various cancer types, EMT *per se* has not been typically observed in prostate cancer. Nevertheless, evidence of EMT-like changes exists during prostate cancer progression, particularly reduced expression of epithelial markers and increased expression of mesenchymal markers. We show that CDK11p58-induced SPDEF degradation reduces expression of the epithelial-specific genes E-cadherin and cytokeratin 18 indicating the CDK11p58-SPDEF axis may play an important role in cancer cell migration and invasion.

A key role for SPDEF in cancer cell migration, invasion and metastasis has emerged, although with opposite conclusions in different cancer types. Whereas, SPDEF overexpression has been shown to inhibit breast cancer cell growth and reduce invasion and migration [5-7, 13, 15], other studies indicate that SPDEF overexpression induces invasion and migration in breast cancer cells [34]. We and others previously demonstrated that loss of SPDEF expression induces morphologic changes, increased migration and invasiveness, and triggers a transcriptional program of genes involved in TGFβ signaling, migration, invasion, adhesion, and epithelial dedifferentiation, establishing SPDEF as a critical regulator of key aspects linked to metastasis in prostate, breast and colon cancer [5, 13-16, 35]. The relevance of SPDEF downregulation for prostate cancer metastasis has been confirmed in xenograft models, supporting our previous conclusions [5, 11, 12]. Most importantly, reduced SPDEF expression.directly correlates with poor outcome in prostate cancer [7-11]. Moreover, reduced SPDEF protein expression during prostate and breast cancer progression has been attributed to dysregulated miRNAs [6]. To decipher the critical steps involved in prostate cancer metastasis, we investigated the relevance of regulation of SPDEF protein stability in invasion and migration of prostate cancer cells and report now a new regulatory mechanism of SPDEF expression and activity that impact on prostate cancer metastasis (Figure 8C). Our migration and invasion data shown here indicate that silencing CDK11p58 and consequent inhibition of SPDEF degradation reduce prostate cancer cell migration. In contrast, CDK11p58 overexpression induced cancer cell migration and invasion, while re-expression of SPDEF or GADD45α, which we demonstrate here to bind CDK11p58 and inhibit its kinase activity, reduced migration and invasion.

We identified CDK11p58 as an interaction partner of GADD45α by co-immunoprecipitation analysis. We corroborated these data by co-immunoprecipitation *in vitro* translation assays, showing that all three members of the GADD45 family interact with CDK11p58. GADD45 proteins have been shown to interact with and inhibit the kinase activity of CDK1 [23, 36]. Here we demonstrate that GADD45α overexpression inhibits CDK11p58 kinase activity and impedes SPDEF degradation mediated by CDK11p58, inhibiting prostate cancer cell migration and invasion. These results clearly demonstrate a new GADD45α mechanistic role during prostate cancer cell migration. The GADD45 gene family (GADD45α, β and γ) has been implicated in regulation of several cellular functions including DNA repair, cell cycle control, apoptosis, senescence and genotoxic stress. Emerging functional evidence implies that GADD45 proteins serve as tumor suppressors in response to diverse stimuli [22] and our results support this notion as GADD45α inhibited SPDEF degradation and thereby reduced migration and invasion. We previously demonstrated that NF-κB-mediated repression of GADD45α and γ is essential for prostate cancer survival [18], and GADD45α expression in prostate cancer is repressed due to methylation [17]. Enhanced GADD45α expression sensitized prostate cancer cells to docetaxel [26]. Some of these critical activities of GADD45α in prostate cancer may be mediated through its regulation of SPDEF protein stability. In summary, the regulation of protein degradation of SPDEF by CDK11p58 may be a crucial factor for inducing prostate cancer cell migration and invasion, with major consequences in prostate cancer metastasis and systemic dissemination. Additionally, GADD45's role in the regulation of CDK11p58 mediating SPDEF degradation introduces a novel activity of GADD45 in cancer cell migration and invasion.

## MATERIALS AND METHODS

### Cell culture

DU145 and HEK 293T cells were obtained from American Type Culture Collection (Rockville, MD) and maintained in Dulbecco's Modified Eagle Medium (DMEM) supplemented with 5% fetal bovine serum (FBS) (Gibco, Invitrogen) and 1% penicillin (5000u/ml)/streptomycin (500μg/ml) (Lonza, Walkersville, MD, USA) at 37°C in a humidified atmosphere of 5% CO2. All cell lines were obtained from ATCC and authenticated *via* short tandem repeat (STR) profiling performed by the company. The experiments were carried out within 6 months of their resuscitation

### Vectors

SPDEF-Flag and SPDEF-myc constructs were obtained by cloning the SPDEF coding sequence into pcDNA3-Flag and pcDNA3-myc vectors, respectively. SPDEF-GST plasmid was obtained by cloning the SPDEF into PGEX-3 vector. Mutations in the SPDEF gene were generated by substitution of serine to alanine using the Quick site-directed mutagenesis II kit (Agilent) and SPDEF-Flag vector as DNA template. The specific locations of the mutated serine residues in the SPDEF protein were: 44/46, 61, 81/82, 220, 231/234, 238/242/243, 308. All the mutated SPDEF sites were sequenced and the mutations confirmed (data not shown). The vectors expressing the CDK11p58-HA and the CDK11p46-myc were kindly donated by Dr. Jianxin Gu, Shanghai Medical Center of Fudan University, P.R China.

### Transfection

Transfections of 5 × 10^5^ cells were carried out with 1μg plasmid DNA using Lipofectamine Plus (Invitrogen). Cells were incubated for 24 or 48 hours in the presence or absence of 1mM MG132.

### *In vitro* transcription/translation

GADD45, SPDEF and CDK11p58 proteins were produced *in vitro* using the TnT^®^ *Quick* Coupled *Transcription*/*Translation* Systems (Promega) according to the manufacterer's instructions and were used immediately for binding and *in vitro* kinase studies.

### Immuoprecipitation and mass spectrometry analysis to identify GADD45 interacting proteins

Immuprecipitations were performed as previously described [18] using anti-FLAG, anti-HA agarose or anti-myc agarose (Sigma) and eluted using Flag peptide (150μg /ml) or HA peptide (100μg /ml) (Sigma). For mass spectrometry studies, the eluted samples were subjected to SDS-polyacrylamide gel, stained by colloidal Coomassie (Bio-Rad). Prominent bands were cut out, digested with trypsin overnight and analyzed by mass spectrometry as described before [18]. The acquired peptide masses were interrogated by ProFound, a protein identification database. For direct interaction studies proteins were produced by *in vitro* transcription/translation kit (Promega) following the manufacturer's protocol and mixed in the presence of the appropriate agarose beads.

### *In vitro* kinase assays

HEK 293T cells were transfected with 1μg of plasmid expressing CDK11p58-HA using Lipofectamine Plus (Invitrogen) in a 6-well plate and incubated for 24 hours. The cells were then washed with PBS and scraped in the presence of lysis buffer (Cell Signaling) and a protease inhibitor cocktail (cOmplete Protease Inhibitor Cocktail, Roche). The lysate was centrifuged with lysis buffer and 200μg of the total protein was immunoprecipitated with anti- HA agarose (Sigma) for 16 hours. The HA-agarose beads were washed 5 times in PBS and once with *in vitro* kinase buffer (50 mM Tris-HCl (pH 7.5), 15 mM MgCl2, 1 mM DDT). The resultant immunoprecipitate was analyzed for CDK11p58 kinase activity in *in vitro* kinase buffer, 50 mM ATP, and SPDEF-GST (2-5 mg/ml). SPDEF phosphorylation was analyzed by 12% SDS polyacrylamide gel and Western blotting using a phospho-serine (pSer) antibody (Chemicon, AB1603). In order to test the inhibitory potential of GADD45, we produced the GADD45 and SPDEF proteins using the TnT^®^ *Quick* Coupled *Transcription*/*Translation* Systems (Promega) and mixed in the *in vitro* kinase reaction.

### GST Pull-down assay

GST or GST fusion proteins were expressed in BL21 cells, and equal amounts of bacterial lysates were incubated with 25μl of glutathione-Sepharose beads for 30 min. The beads were then washed three times with GST Lysis buffer (20 mM Tris-Cl (pH 8.0), 200 mM NaCl, 1 mM EDTA (pH 8.0), 0.5% Triton X-100), and incubated with 5μl of *in vitro* translated (TnT^®^ *Quick* Coupled *Transcription*/*Translation* Systems - Promega) proteins in protein binding buffer for 16 hours at 4°C. The beads were then washed five times with binding buffer and boiled in SDS sample buffer.

### Immunofluorescence

Formaldehyde-fixed, permeabilized cells cultured on glass coverslips were incubated with primary antibodies, anti-myc-Cy3 (Sigma), anti-Flag produced in rabbit (Sigma), and anti-HA-FITC (Sigma) for 1 hour at 37°C, followed by incubation with secondary anti-rabbit-Cy5 (KPL) and DAPI. Cells were analysed on a confocal laser scanning microscope (Karl Zeiss).

### Generation of stable cell lines

DU145 CDK11p58 knock-down (DU145CDK11p58−/−) cells were generated by transducing DU145 cells with the MISSION Lentiviral Transduction Particles (Sigma-Aldrich) encoding siRNA against CDK11p58 (clone ID: TRCN0000006992 - target sequence: CGTATAGAAGAGAAGACTCAA) and selected using 10μg /ml puromycin (Invitrogen). DU145 cells overexpressing the CDK11p58 protein (DU145CDK11p58+/+) were generated by transfection with the CDK11p58-HA expression vector and selected with 1 mg/ml G418 (Sigma-Aldrich).

### Real-time PCR

Total RNA was harvested using QIAshredder (Qiagen, Valencia, CA) and the RNeasy mini kit (Qiagen). Real time PCR analysis of Cytokeratin 18 and E-cadherin was performed as described [5].

### Invasion and migration assays

Cell migration and invasion assays were performed as described [5] using a modified transwell chamber migration assay and invasion assay matrigel-coated membrane (BD Biosciences Bedford, MA).

### Wound healing assay

Wound healing assays were carried out in DU145 and DU145-CDK11p58−/− cells. A scratch was created in near-confluent cell cultures using the tip of a P-1000 pipetman. The plates were photographed at 0, 8, 16 and 20h using microscope digital camera (Olympus sc30) and Analysis GetIt software at three different points down the length of the scratch. The images were then analysed using WCIF Image J software.

### Western blot analysis

Western blot analysis was performed as described [5, 18]. The primary antibodies used for Western blot analysis were anti-phospho-serine (Chemicon), β-actin (Santa Cruz), anti-Flag-HRP (Sigma), anti-HA-HRP (Sigma), anti-myc-HRP (Sigma), anti-SPDEF (Santa Cruz), and anti-CDK11p58 (Santa Cruz).

### Statistical analysis

Statistical analysis was performed using Student's *t*-test was used to evaluate the statistical difference among the groups compared to the control.

## SUPPLEMENTARY MATERIAL FIGURES


