# Supplementary material for: GADD45α and γ interaction with CDK11p58 regulates SPDEF protein stability and SPDEF-mediated effects on cancer cell migration
Source: Oncotarget. 2016 Feb 12;7(12):13865–79. doi: 10.18632/oncotarget.7355 (PMC4924684; doi:10.18632/oncotarget.7355)
Supplement: Supplementary file 1 [file oncotarget-07-13865-s001.pdf]

GADD45α and γ interaction with CDK11p58 regulates SPDEF protein stability and SPDEF-mediated effects on cancer cell migration

Supplementary Material

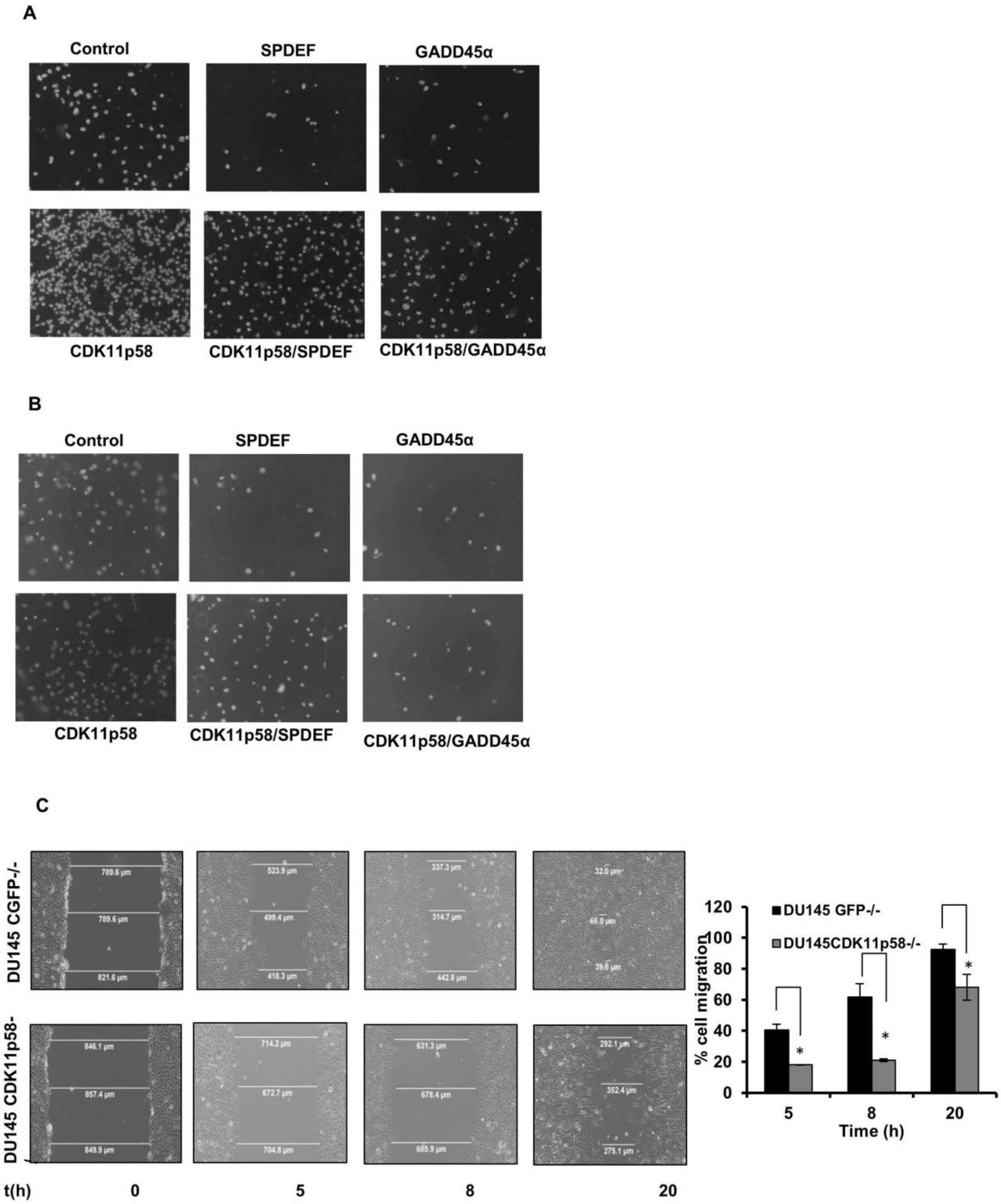

Supplementary Figure S1. Biological relevance of SPDEF regulation by CDK11p58. CDK11p58 affects prostate cancer cell migration and invasion. DU145 was transfected with SPDEF-Flag, GADD45α-Flag, CDK11p58-HA, CDK11p58-HA/SPDEF-Flag, CDK11p58-HA/GADD45 α-Flag or mock transfected. (a) Migration and (b) invasion were measured using transwells 24h post-transfection. Cell were fixed and stained, and 3-5 random microscopic fields were counted. Values shown are mean±s.d. from a representative experiment. (c) Wound healing assay. Wounds were measured in three different positions at 0, 5, 8 and 20 h and mean distance of control set as 100%. (D) Graph representation of wound healing assay. Student-t test was used to evaluate the statistical difference among the groups compared to the control * indicates statistical significance with p-value <0.05.

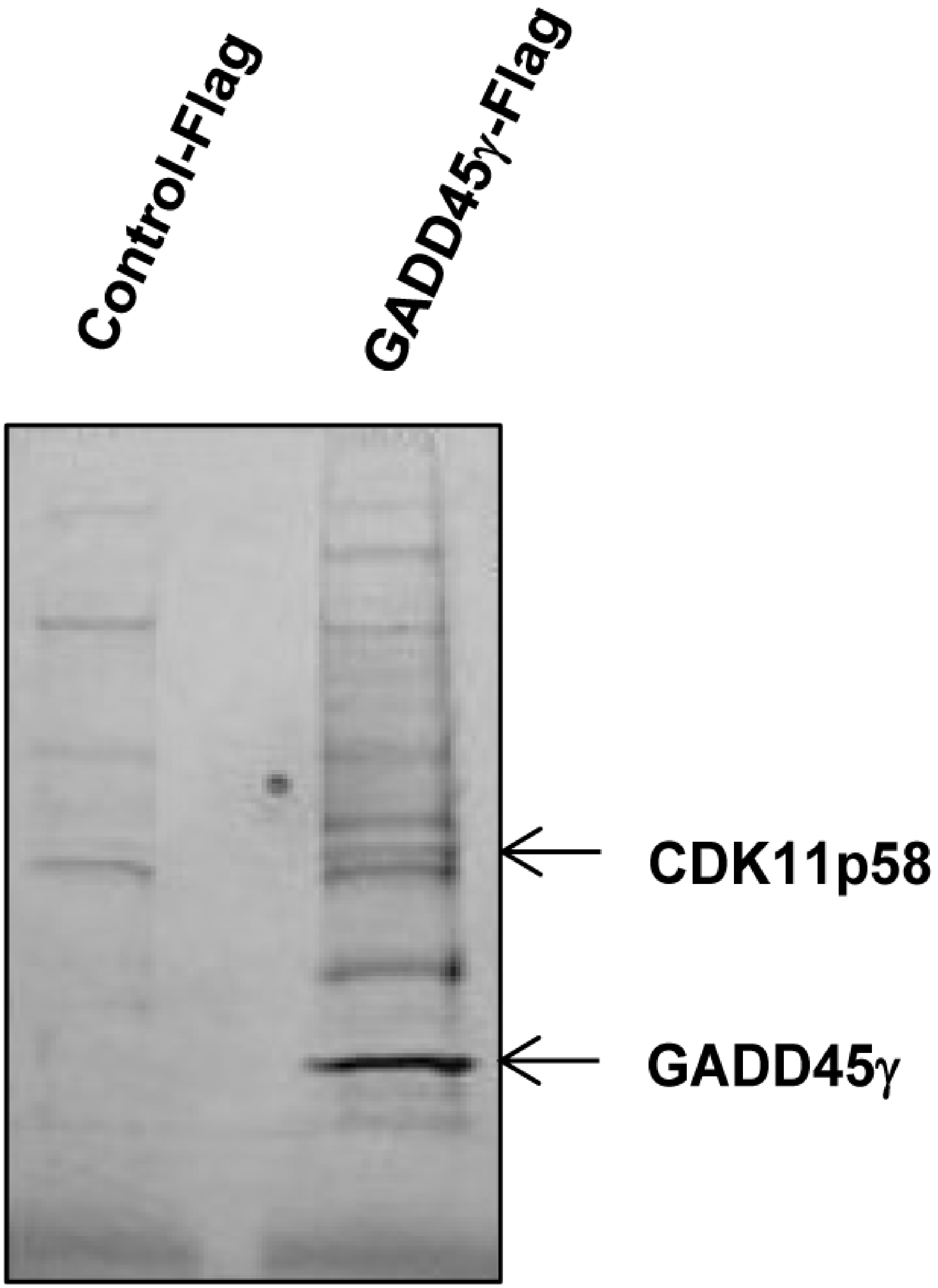

Supplementary Figure S2. Interaction of SPDEF, CDK11p58 and GADD45. Interaction of GADD45 γ and CDK11p58 *in vivo*. GADD45α-Flag or Control-Flag vectors were transfected into HEK 293T cells and whole cell extracts were prepared 24h post-transfection. The samples were immunoprecipitated with anti-FLAG monoclonal antibody (mAb) and proteins were analysed by SDS-PAGE, cut out, and trypsinized followed by SELDI-TOF-MS. The acquired peptide masses were interrogated by the ProFound database

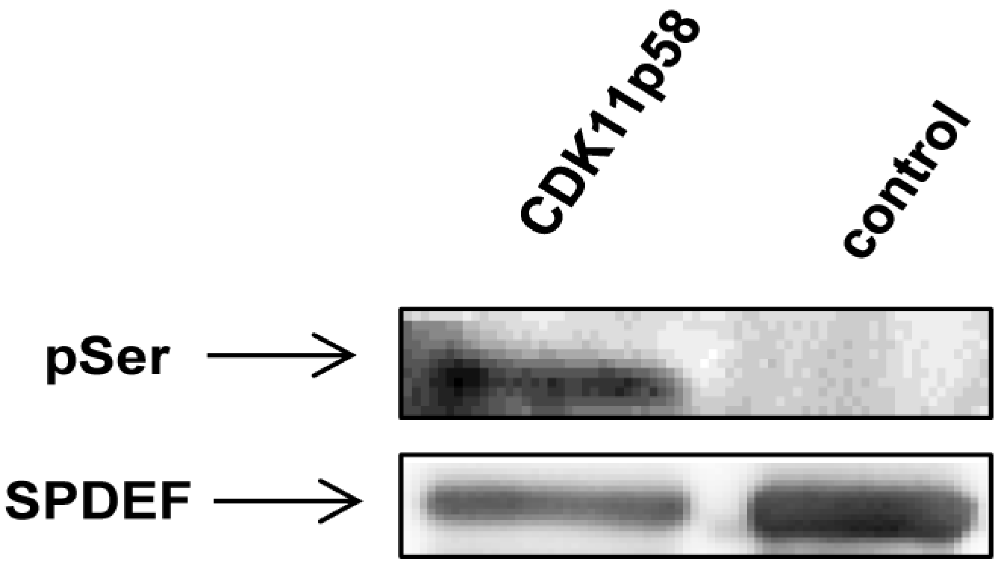

Supplementary Figure S3. GADD45 inhibits the kinase activity of CDK11p58. SPDEF-flagwas produced *in vitro* by TNT® *Quick* Coupled transcription/translation system (Promega) and combinations of SPDEF, mock or SPDEF plus cdk11p58 proteins were subjected to *in vitro* kinase assay mediated by CDK11p58. PDEF was detected using anti-flag and phosphorylated PDEF was detected with anti-serine antibody.

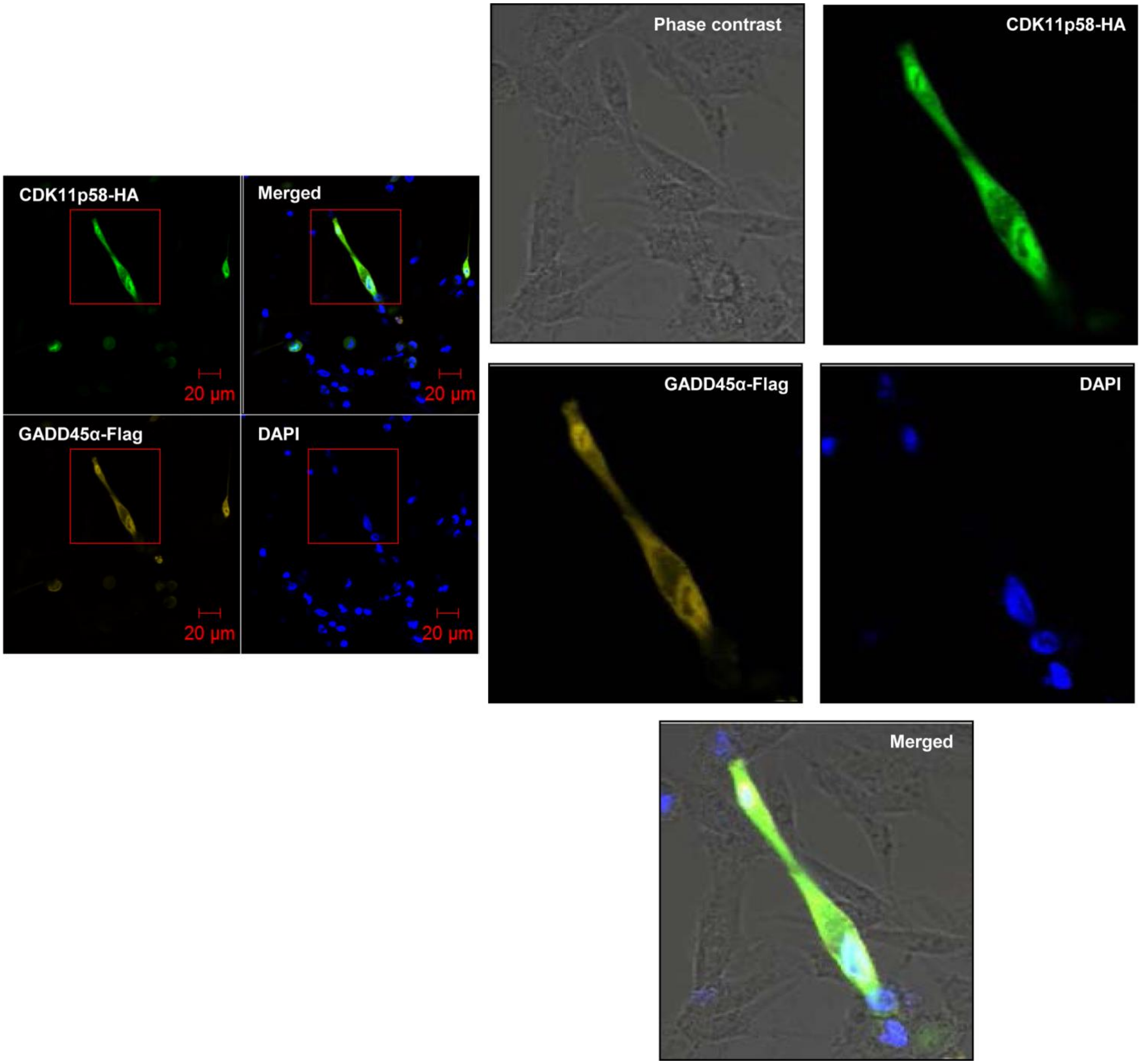

Supplementary Figure S4. Cellular localization of GADD45 and CDK11p58. DU145 cells were transfected with GADD45 α -Flag and CDK11p58-HA. 24 hours post transfection, cells were fixed by 70% ethanol, and incubated for 1 hour with antibodies against HA-FITC and against Flag produced in rabbit, following which the cells were incubated for another hour with antibody against rabbit conjugated with Cy5 and DAPI. The cells were analyzed by Confocal Laser Scanning Microscope (Carl Zeiss).
